# Four decades of the Graf method in screening for developmental dysplasia of the hip (part I): Rightly the gold standard or of dubious benefit?

**DOI:** 10.3389/fped.2022.1002696

**Published:** 2022-11-18

**Authors:** Robert Ossendorff, Sonja Placzek, Rahel Bornemann, Sebastian G. Walter

**Affiliations:** ^1^ Department for Orthopedic Surgery and Traumatology, University Hospital Bonn, Bonn, Germany; ^2^ Medical Service of the Health Funds (MDK), Cologne, Germany; ^3^ Department for Orthopedic Surgery and Traumatology, University Hospital Cologne, Cologne, Germany

**Keywords:** Graf, developmental dysplasia of the hip, hip, ultrasound, dysplasia

## Abstract

**Introduction:**

The method of infant hip sonography according to Graf is used for general hip screening in Serbia, Austria, and Germany and is considered the gold standard. In other countries, such as the USA, however, it is not well accepted and is claimed to lead to high costs and overtreatment. The aim of this study was to investigate how many of the mentioned sources in a recent review article contained sonograms that met the quality criteria as taught in Graf's ultrasound courses.

**Methods:**

A systematic review published by Sakkers et al. was analyzed in terms of addressing the quality criteria of Graf’s method. Studies that were suitable by title, abstract, manuscript, that contained an image of sonographic hip examination, and that were accessible were included into analysis.

**Results:**

Within the described review, there were 22 papers on the Graf method. Of these, 10 contained hip sonograms and were applicable for final analysis. The quality criteria according to Graf were not fulfilled within 5 of these 10 papers. Within these papers, there are examples of schematic sonograms that do not correspond with the quality criteria either.

**Conclusion:**

Skepticism regarding the Graf method may be based on user errors and insufficient application of the Graf quality assessment algorithm, which results in high intra- and inter-observer variations. Based on these findings, a working group was initiated to evaluate further work according to the same procedure (currently approximately 130 papers).

## Introduction

The method of infant hip sonography according to Graf is established for general hip screening in Serbia, Austria, and Germany and is considered the gold standard.

Among doctors trained in Germany and working in the field of pediatrics, pediatric orthopedics, or radiology, there is not the slightest doubt about the importance and validity of the general ultrasound screening of the infant hip according to Graf. Accordingly, a statement such as “(…) improvement (…) is still under debate” (1) in the central organ of the German medical profession led to indignant letters to the editor from committed colleagues (2). On the other hand, the question arises why, approximately 40 years after the description of the Graf method and more than a quarter of a century (1996 in Germany) after the establishment of a general hip screening, the evaluation of the method in the international literature sometimes appears to be very different from studies from Germany or Austria.

The authors therefore asked themselves whether the different evaluation of the Graf method is due to a different definition/application. To test this assumption, the following hypothesis was made:

Do the papers listed in a current and international systematic review (3) correspond to the quality criteria of the Graf Method as taught in courses?

The aforementioned review paper states verbatim:

“The fact that even the unstable and dislocated hips do relatively well without treatment in a substantial percentage probably contributes to the fact that all studies on US screening of hips for detection of relevant DDH in order to improve outcomes of treatment are rated as substantially underpowered” (3).

## Methods

**Figure 1:**
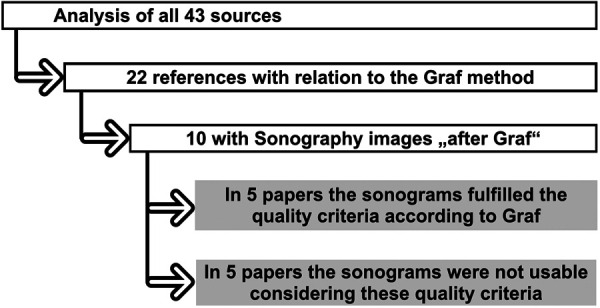
Overview of systematic evaluation of the original articles used by Sakkers et al. (3). 10 articles included sonograms, only 5 articles fulfilled the quality criteria according to Graf.

**Figure 2:**
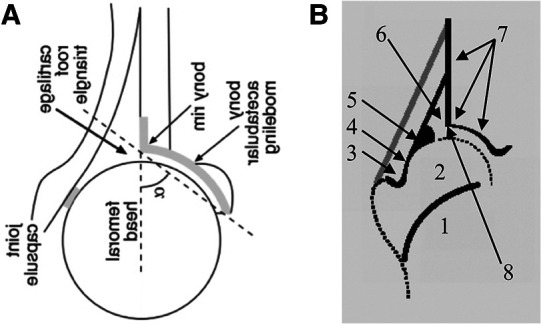
Schematic of the systematic evaluation of hip sonograms of different original articles (**A**). The original diagram from (5) was rotated by 90° and mirrored around the transversal axis for better clarity (ultrasound from the left, upright image). (**B**) Scheme of anatomical identification (Checklist I) from a course manual for the ultrasound course according to Graf (6). The numbers describe: 1, ChB (chrondro-osseous border); 2, femoral head; 3, synovial fold; 4, joint capsule; 5, labrum; 6, cartilage; 7, bony roof; 8, bony rim (turning point).

To check the quality criteria, all the original papers cited in the review were examined and those that dealt with ultrasound screening according to Graf were selected. The selected papers were searched (Figure 1) for ultrasound images and these were examined by two independent doctors experienced in sonography with regard to the quality criteria after Graf: Checklist I (anatomical identification) and Checklist II (usability check) (Figure 2) (7).

The alignment of sonograms in either lying or standing upright positions and performance of the ultrasound from either the left or the right side was not taken into account as error.

## Results

The analysis of the 43 listed sources is shown in Figure 1. Of these 43 sources, 22 dealt with the Graf method. Of these, 10 contained sonograms “according to Graf” (Table 1).

**Table 1:** Overview on final 10 analyzed studies.

Author	Year	Error	Title
Castelein, RM	1992	Checklist I	**Natural history of ultrasound hip abnormalities in clinically normal newborns.**
Holen, KJ	1994		Ultrasound screening for hip dysplasia in newborns.
Marks, DS	1994		Routine ultrasound screening for neonatal hip instability. Can it abolish late-presenting congenital dislocation of the hip?
Rosendahl, K	1996		Developmental dysplasia of the hip: prevalence based on ultrasound diagnosis.
Terjesen, T	1996	Tilting error	**Hip abnormalities detected by ultrasound in clinically normal newborn infants.**
Lorente Moltó, FJ	2002		Three-year prospective study of developmental dysplasia of the hip at birth: should all dislocated or dislocatable hips be treated?
Roovers, EA	2005		The natural history of developmental dysplasia of the hip: sonographic findings in infants of 1-3 months of age
Chen, HW	2010	Tilting error	**Natural progression of hip dysplasia in newborns: a reflection of hip ultrasonographic screenings in newborn nurseries.**
Rosendahl, K	2010	Checklist I	**Immediate treatment versus sonographic surveillance for mild hip dysplasia in newborns.**
Laborie, LB	2014	Checklist I	**Selective ultrasound screening for developmental hip dysplasia: effect on management and late detected cases. A prospective survey during 1991–2006.**

Highlighted studies demonstrated sonograms that did not correspond to the Graf criteria.

Five articles showed sonograms that met the quality criteria according to Graf (Checklists I and II). The other five papers contained at least one or more sonograms that did not meet the quality criteria.

In the five papers that did not meet the quality criteria, the anatomical identification specified in Checklist I was impossible in the sonograms of three papers. In the other two papers, the sonograms did not meet the usability check (Checklist II). Tilting errors were present.

Surprisingly, in the two most recent studies (from 2010 and 2014) (4, 5), in which one or more sonograms did not fulfil the quality criteria according to Graf, schematic representations of the anatomical structures were also found, which also did not correspond to Checklist I (anatomical identification).

Figure 2 shows an example of the analysis of the most recent of the 10 cited papers with sonograms.

The evaluation of the sonograms was not accurate according to Graf. For instance, no chondro-osseous border was shown in the schematics of both studies (4, 5).

A comparison of the three schematic figures shows that with regard to point 1 (ChB) and point 3 (Synovial Fold), Checklist I is not fulfilled.

## Discussion

It is unclear whether the work by Sakkers et al. analyzed in this paper can be considered representative of the international literature. However, with its 22 cited papers dealing with the Graf method, it can certainly be seen as a representative cross-section. Half of the original papers with sonograms did not meet the defined quality criteria according to Graf, at least in one image. This fact suggests that these quality criteria are not given the necessary importance in the teaching of this examination method. This coincides with the diagrams, some of which do not contain anatomical structures that meet the quality criteria.

However, this shows that the insufficient sonograms are not a result of cursorily performed sonographic examinations, but a deficit of knowledge about the method.

The fact that the work by Graf cited by Sakkers et al. dates from 1980 should be seen in the same context. At that time, the ultrasound method taught today according to Graf had not yet been developed in its current form and the quality criteria (Checklist I—anatomical identification; Checklist II—brewability test), which have now been obligatory for decades, had not yet been defined.

## Conclusion

The statements made by Sakkers et al., as far as they are based on data of the Graf method, must be doubted, as some of the underlying studies do not consider the defined criteria of this method. Based on these findings, a working group was initiated to evaluate the sonograms of further papers (currently approximately 130 papers, Part II).

## Data Availability

The original contributions presented in the study are included in the article/Supplementary Material. Further inquiries can be directed to the corresponding author/s.
